# New insights on key genes involved in drought stress response of barley: gene networks reconstruction, hub, and promoter analysis

**DOI:** 10.1186/s43141-020-00104-z

**Published:** 2021-01-06

**Authors:** Seyedeh Mehri Javadi, Zahra-Sadat Shobbar, Asa Ebrahimi, Maryam Shahbazi

**Affiliations:** 1 Department of Biotechnology and Plant Breeding, Science and Research Branch, Islamic Azad University, Tehran, Iran; 2 Department of Systems Biology, Agricultural Biotechnology Research Institute of Iran (ABRII), Agricultural Research, Education and Extension Organization (AREEO), Karaj, Iran; 3 Gorgan University of Agricultural Sciences and Natural Resources, Gorgan, Iran

**Keywords:** Barley, Drought stress, Biological networks, Hub gene, Promoter analysis

## Abstract

**Background:**

Barley (*Hordeum vulgare* L.) is one of the most important cereals worldwide. Although this crop is drought-tolerant, water deficiency negatively affects its growth and production. To detect key genes involved in drought tolerance in barley, a reconstruction of the related gene network and discovery of the hub genes would help. Here, drought-responsive genes in barley were collected through analysis of the available microarray datasets (− 5 ≥ Fold change ≥ 5, adjusted *p* value ≤ 0.05). Protein-protein interaction (PPI) networks were reconstructed.

**Results:**

The hub genes were identified by Cytoscape software using three Cyto-hubba algorithms (Degree, Closeness, and MNC), leading to the identification of 17 and 16 non-redundant genes at vegetative and reproductive stages, respectively. These genes consist of some transcription factors such as *HvVp1*, *HvERF4*, *HvFUS3*, *HvCBF6*, *DRF1.3*, *HvNAC6*, *HvCO5*, and *HvWRKY42*, which belong to AP2, NAC, Zinc-finger, and WRKY families. In addition, the expression pattern of four hub genes was compared between the two studied cultivars, i.e., “Yousef” (drought-tolerant) and “Morocco” (susceptible). The results of real-time PCR revealed that the expression patterns corresponded well with those determined by the microarray. Also, promoter analysis revealed that some TF families, including AP2, NAC, Trihelix, MYB, and one modular (composed of two HD-ZIP TFs), had a binding site in 85% of promoters of the drought-responsive genes and of the hub genes in barley.

**Conclusions:**

The identified hub genes, especially those from AP2 and NAC families, might be among key TFs that regulate drought-stress response in barley and are suggested as promising candidate genes for further functional analysis.

## Background

Barley (*Hordeum vulgare* L.) is one of the most important cereals in the world [1]. The plant is ecologically adaptable to a wide range of environments and generally has higher drought tolerance compared to other cereals. Barley is usually regarded as a suitable model for studying abiotic stress because of high genomic diversity and unique physiological/morphological properties [2]. Drought stress is among the strongest environmentally effective parameters limiting barley’s growth and productivity [3, 4]. Since climate change has reduced annual amounts of rainfall and increased the temperature in most areas [5–7], drought tolerance is considered as a complex character that includes a series of physiological, morphological, and biochemical changes in the plant [8]. These can include earliness, reduced leaf area, leaf sprain, increased efficiency of the root system, reduced tillering, and accretion of osmoprotectants [9]. Extensive studies have been conducted to identify the genes involved in each of these responses in barley [10–17].

To better understand drought tolerance and its mechanisms, the identification of DEGs in stress conditions could be a preliminary step, while reconstructing the related network and detecting the key genes would be the next necessary steps. By completing many genome projects, researchers have turned their attention from investigating individual genes to studying their interaction networks, since the components do not operate independently inside the cell, and their performance and features are defined in operation with other elements. The discovery and analysis of cell biological processes is one of the main objectives in the post-genomic period [18]. Cellular processes are adjusted using the interaction between different molecules such as protein, DNA ,and metabolites [19, 20]. Regarding the crucial role of regulatory elements in plant responses to drought stress [21], promoter analysis is a powerful method to understand the mechanism [21]. Currently, many studies indicate that the barley transcription factor is involved in drought tolerance. Sazegari et al. [22] reported that *ERF/AP2*, *C2C2-Dof*, and *bHLH* transcription factor families are regulatory components of the transcriptional cascade involved in priming-induced tolerance. The expression of the gene for the barley MYB transcription factor, *HvMYB1*, was reportedly upregulated in roots and leaves by drought, which reduced stomatal conductance, enhanced proline content, and reduced ROS levels and catalase activity [23]. Ju et al. [24] reported that under drought conditions the *VvNAC17*-overexpression lines had lower malondialdehyde and H_2_O_2_ contents, but higher peroxidase, superoxide dismutase, catalase activity, and proline content. The WRKY belongs to an important plant-specific transcription factor family which is involved in response to environmental stresses. Analyzing the available microarray data revealed eight candidate WRKY genes which were upregulated under drought and salinity stresses compared to the optimum conditions at seedling stage in barley [25]. In the current research, we attempted to collect highly drought-responsive genes in barley through analysis of all the available related microarray data [10–14]. Then, the gene regulatory and protein-protein interaction (PPI) networks were reconstructed using the resultant gene list. To find the key genes regulating the drought stress response, a hub analysis was performed. Moreover, promoter analysis revealed the most common transcription factor families that have binding sites in the promoters of the highly drought-responsive genes. Finally, real-time PCR was used for validating some of the most promising candidate genes.

## Methods

### Identification of drought-inducible genes in barley

All the available microarray data (up to the time of the study) on barley (*Hordeum vulgare*) regarding its responses to drought stress (GSE3170, GSE6990, GSE15970, and GSE17669 Datasets) were downloaded at https://www.ncbi.nlm.nih.gov/geo/ (Table 1). Data normalization was done using the RMA algorithm by Expression console software, Version 1.3.1 (https://www.thermofisher.com/.../affymetrix-expression-console-software.html). Differentially expressed genes with a fold change of ≥ 5 and ≤ − 5 and adjusted *p* value ≤ 0.05 were identified using FlexArray software (ver1.6.3) (http://www.affymetrix.com/products/software/compatible/index.affx). Redundant genes that were obtained from different microarray datasets were removed. The ortholog of these non-redundant genes in Arabidopsis (*Arabidopsis thaliana*) was identified using BLASTx (https://blast.ncbi.nlm.nih.gov/Blast.cgi) and BLASTn [28]. These orthologous genes in Arabidopsis were used for network reconstruction.

**Table 1:** The microarray data series used in this study

Series	Technology	Genotype	Tissue	Developmental Stage	Reference
GSE3170	GeneChip arrays- Affymetrix	Barke, Morex and Stepoe	Seedlings	Vegetative stage/seedlings	Cui et al. [26]
GSE6990	GeneChip arrays- Affymetrix	Morex	Crown	Vegetative stage/seedlings	Tommasini et al. [27]
GSE15970	GeneChip arrays- Affymetrix	Martin, HS41-1, Moroc9-75	Flag leaves	Reproductive stage/post anthesis	Guo et al. [12]
GSE17669	GeneChip arrays- Affymetrix	Morex	Spikes (Lemmas, paleas, awns, and seeds)	Reproductive stage/grain-filling	Abebe et al. [13]

### Reconstruction of genes and protein-protein interactions (PPIs) networks and the hub analysis

The mentioned list of genes was analyzed in the web-based application of STRING ver.10 [29] (http://string-db.org), and the protein-protein interactions list was prepared. Cytoscape (Ver 3.4) and plugin of Cyto-Hubba were used for drawing the protein-protein interaction network and for identifying highly connected protein nodes (hubs) [30]. Three computational algorithms of Cyto-Hubba named Degree, Closeness, and MNC were used for detecting hub genes (10 nodes with the most interactions).

### Gene Ontology enrichment analysis of DEGs

Gene Ontology (GO) enrichment was analyzed at http://bioinfo.cau.edu.cn/agriGO. Annotations were made for GO terms based on biological processes (BP), cellular components (CC), and molecular function (MF) [31]. The significance of the GO terms was tested through the Fisher’s exact test (*P* < 0.05) [32].

### Promoter analysis

Promoter sequences of the genes (identified orthologs in Arabidopsis) were derived using the Gene2Promoter program available in Genomatix Software Suite (https://www.genomatix.de). The promoter areas of the genes were defined as 1000 and 200 bp (upstream and downstream, respectively) of the transcription initiation site in the intended gene. Common TFs and framework analyses were used respectively for detecting the common transcription factors and common modules, which were present in 85% of the promoters. The similarity level of the core was considered to be 1 in the conducted analysis, and the level of similarity matrix was set on the optimized option.

### Plant material and experimental treatment

The seeds of two spring barley genotypes (i.e., “Morocco” as a drought-susceptible and “Yousef” as a drought-tolerant variety of barley) were used in experiments laid out as RCBD for the two treatments (well-watered and drought-stressed) and three replicates in a greenhouse. The plants were cultivated in well-watered settings until flowering, and the drought treatment begun at this time by withholding irrigation. Samplings were performed randomly among the main stems with the same height, i.e., 21 days after anthesis (DAA). The samples were subject to rapid freezing in liquid nitrogen and were kept at -80 °C for the experiments.

### Real-time PCR

To validate the bioinformatic data, four hub genes (*HvDRFI.3*, *HvCBF6*, *HvCO5*, and *HvWRKY42*) were selected. Total RNA was extracted from penultimate tissues of the barley genotypes in three biological replicates using Trizol reagent as instructed by the producer. To confirm the removal of genomic DNA, RNA samples were subjected to RNase-free DNase treatment (Promega, USA). cDNA was synthesized by the iScript kit. The qRT-PCR was carried out by a LightCycler® 96 Real-time PCR System and iQ Syber Green Supermix kit as per the company’s instructions. The gene-specific primers (Supplementary Table 9) were designed using Oligo 7.0. The qRT-PCR was implemented for three replications of both control and drought-treated samples from both barley genotypes with a LightCycler® 96 Real-Time PCR System and SYBR Premix EX Taq II as described in the producer’s guidelines. Relative mRNA abundance (in fold change) was measured via the delta-delta Ct (ΔΔCt) technique [33] after normalization of the Ct value for individual genes versus Actin (AY145451.1) as the endogenic control. Fold change was estimated using the REST software to analyze qPCR data (based on the Pfaffl method) [34].

## Results

### Identification of drought-responsive genes in barley

A total of 250 differentially expressed genes (DEGs) (5 ≤ fold change ≤ − 5) were obtained, while 134 and 116 of which were upregulated and downregulated, respectively, in drought conditions based on the microarray data analysis (Table 1) after removal of the duplicated genes (Supplementary Tables 1 and 2). We further classified the DEGs into vegetative and reproductive organs including 215 and 35 genes, respectively.

### Reconstruction of genes and PPIs networks, in addition to the hub analysis

**Fig. 1:**
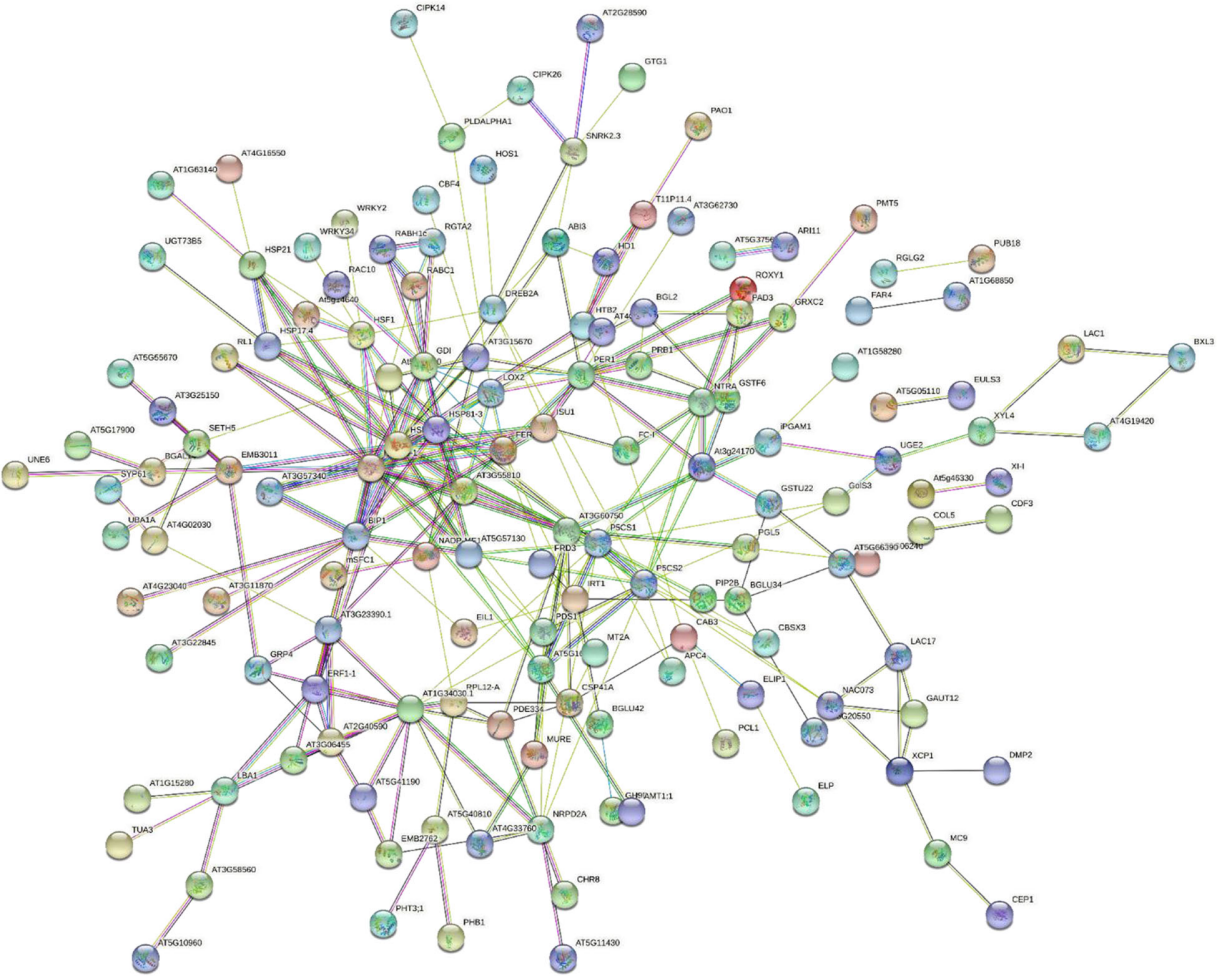
Network of the genes with differential expression of ≥ 5 and − 5 ≥ at drought stress conditions plus their known neighbors in vegetative and reproductive organs of barley based on the microarray data using web based String software

The gene and PPI networks were reconstructed (Fig. 1 and Supplementary Fig. 1). The hub analysis led to the identification of 17 and 16 non-redundant genes with the most interactions being in vegetative and reproductive organs, respectively (Supplementary Tables 3 and 4). A significant proportion of the hub genes was related to regulatory processes such as transcription and expression regulation (e.g., *HvWRKY42*, *HvVP1*, *HvCBF6*, and *DRF1.3*), which belong to transcription factor families.

### Gene Ontology enrichment analysis of the DEGs

**Fig. 2:**
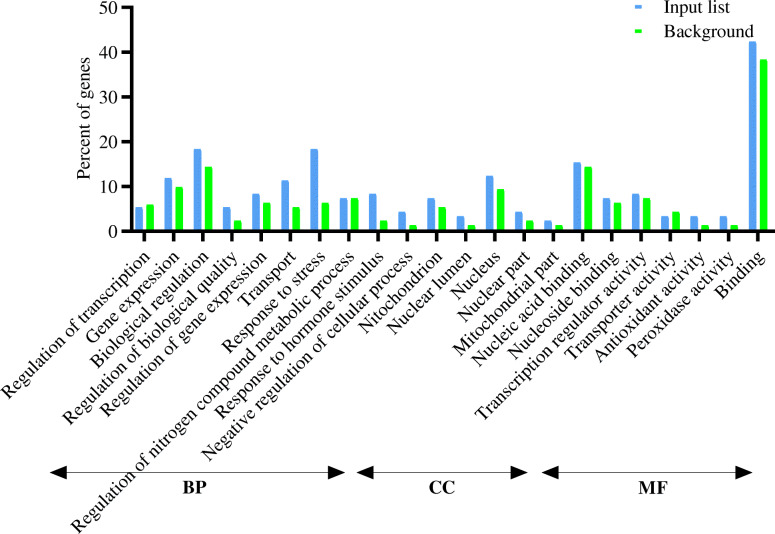
Gene Ontology enrichment analysis (BP, biological processes; CC, cellular components; and MF, molecular function) of differentially expressed genes (≤ 5 and ≥ − 5) at drought stress conditions in vegetative stage barley based on microarray data using agriGO

**Fig. 3:**
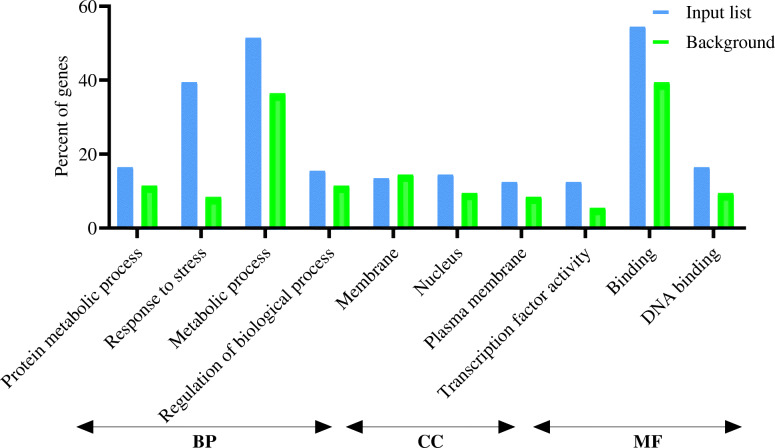
Gene Ontology enrichment analysis (BP, biological processes; CC, cellular components; and MF, molecular function) of differentially expressed genes (≤ 5 and ≥ − 5) at drought stress conditions in reproductive stage barley based on microarray data using agriGO

**Fig. 4:**
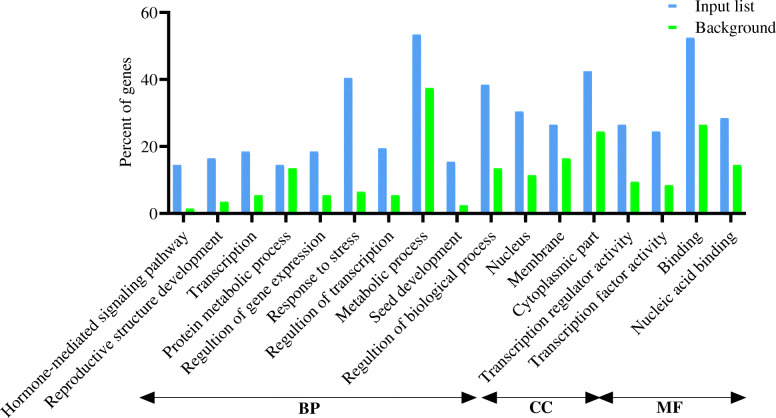
Gene Ontology enrichment analysis (BP, biological processes; CC, cellular components; and MF, molecular function) of the determined hub genes at drought stress conditions in vegetative and reproductive stage barley based on microarray data using agriGO

The SEA was utilized for testing the GO enrichment according to the 250 DEGs in vegetative and reproductive organs and hub genes. Several items were detected as predominant terms, i.e., metabolic processes, stress responses, responses to hormonal stimuli, hormone-facilitated signaling pathways, transportation, protein metabolic processes, gene expression, transcription, and regularization of BP. The most significant MF terms were binding, nucleotide-binding, and transcription activator activity. Concerning the CC ontology, the enriched term was the nucleus (Figs. 2, 3, and 4).

### Promoter analysis

**Fig. 5:**
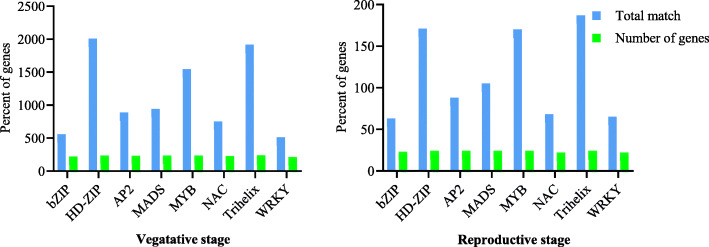
Transcription factor families with binding sites in more than 85% (*P* value < 0.05) of the drought-responsive genes. Blue bar, the number of binding sites of these transcription factors predicted in the promoters of the DEGs; green bar, the number of genes having binding sites of these transcription factors in the promoters

**Fig. 6:**
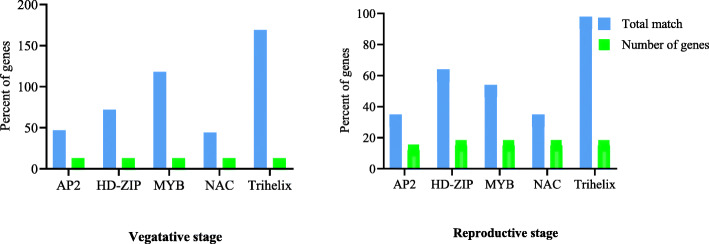
Transcription factor families with binding sites in more than 85% (*P* value < 0.05) of the hub genes. Blue bar, the number of binding sites of these transcription factors predicted in the promoters of the hub genes; green bar, the number of hub genes having binding sites of these transcription factors in the promoters

Based on the results, some transcription factor families including AP2, bZIP, HD-ZIP, MYB, NAC, MADS, Trihelix, and WRKY had binding sites in more than 85% (*P* value < 0.05) of the drought-responsive genes (Fig. 5 and supplementary Table 5) and the hub genes (Fig. 6 and supplementary Table 6). Interestingly, the number of binding sites in these transcription factors were predicted and significantly high in the promoters of the differentially expressed genes and in the hub genes (Figs. 5 and 6 and supplementary Tables 5 and 6). For instance, 1945 and 1854 putative binding sites were found for TFs with HD-ZIP and Trihelix domains in the 250 drought-responsive genes.

### Cooperative modules analysis

One module composed of two HD-ZIP transcription factors (Table 2) and was identified in 85% of the promoters (about 688 out of 212 DEGs). Also, six cooperative modules, i.e., DOF-C2C2 zinc finger domain, Trihelix domain, AT-hook, HD-ZIP, MYB domain, and Heterotrimeric were identified in the hub genes (Table 3). Besides, known modules (i.e., previously reported modules that are available in the database) were also searched in the promoter of the DEGs. According to the results, the binding site of an additional 261 modules in the DEGs (Supplementary Table 7) and 23 modules in the hub genes were identified (Supplementary Table 8). The most common module being identified among the drought-responsive genes was the GATA_HNF1-01 module with the C2C2 zinc finger domain transcription factor from the GATA binding factors family and the Hepatic Nuclear Factor from the homeodomain family which had 19 binding sites in 19 genes (Supplementary Table 7). Also, the most common module being identified in the promoter of hub genes was the GTBX_GTBX_01 Module, with six binding sites (Supplementary Table 8).

**Table 2:** Cooperative module found in 85% of the drought responsive promoters

Module	Elements	Domain	Strand	Matrix similarity	Distance to next element	Common to	*p* value
1	P$AHBP	HD-ZIP	−	0.85 (min. 0.85)	9–18 bp	688 matches in 212 seq. (85%), 532 non-overlapping	1.44434e−15
	P$AHBP	HD-ZIP	+	0.85 (min. 0.85)	----------

**Table 3:** Cooperative modules found in the promoter of the hub genes

Module	Elements	Domain	Strand	Matrix similarity	Distance to next element	Common to	*p* value
1	P$DOFF	DOF-C2C2 zinc finger domain	+	0.85(min. 0.96)	164–173 bp	19 matches in 15 seq. (88%), 18 non-overlapping	1.40355e−06
	P$GTBX	Trihelix domain	+	0.85(min. 0.86)			
2	P$HMGF	AT-hook	+	0.85(min. 0.88)	178–187 bp	20 matches in 15 seq. (88%), 17 non-overlapping	1.61627e−06
	P$DOFF	DOF-C2C2 –zinc finger domain	+	0.85(min. 0.96)			
3	P$AHBP	HD-ZIP	−	0.85(min. 0.86)	59–68 bp	22 matches in 15 seq. (88%), 19 non-overlapping	1.32362e−05
	P$SEF4	structure of DNA-binding domain not specified	−	0.85(min. 0.91)			
4	P$AHBP	HD-ZIP	+	0.85(min. 0.86)	173–182 bp	34 matches in 16 seq. (94%), 29 non-overlapping	2.11074e−05
	P$CAAT	heterotrimeric transcription factor	−	0.85(min. 0.86)			
5	P$AHBP	HD-ZIP	+	0.85(min. 0.86)	154–163 bp	28 matches in 15 seq. (88%), 24 non-overlapping	2.29321e−05
	P$GTBX	Trihelix domain	+	0.85(min. 0.85)			
6	P$HMGF	AT-hook	−	0.85 (min. 0.90	77–86 bp	22 matches in 15 seq. (88%), 22 non-overlapping	2.37645e−05
	P$MYBL	MYB domain	−	0.85(min. 0.85)			

### Validation of the candidate genes

**Fig. 7:**
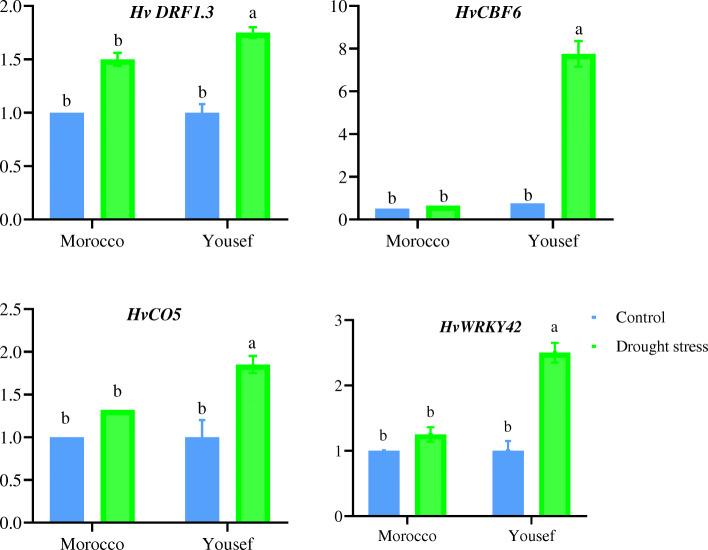
The expression pattern of four promising candidate genes (*HvDRFI.3*, *HvCBF6*, *HvCO5*, and *HvWRKY*42) under drought conditions in *H. vulgare* (Yousef, the tolerant, and Moroco, the sensitive cultivars)

The expression pattern of four promising candidate genes (i.e., *HvWRKY42, HvCO5, HvCBF6*, and *HvDRF1.3*) which were found as hubs through the reconstruction of PPI networks and the hub analysis was evaluated by real-time PCR. Based on the results, the transcript levels of these genes were significantly increased under drought conditions in the “Yousef” genotype, whereas the *HvDRF1.3* gene was only induced in another genotype (Fig. 7). The results of real-time PCR showed that the expression pattern of the four genes was in good agreement with the results obtained by microarray analysis.

## Discussion

In the present study, a total of 250 DEGs were obtained. Tommasini et al. [27] reported that in crown tissues of plants (5 ≤ fold change ≤ − 5), 3330 genes were differentially expressed between the control group and drought stressed samples. A total of 144, 66, and 53 genes were differentially expressed (*P* < 0.0001, |log2-fold| value ≥ 2) between drought-stressed and control plants of Martin, HS41-1, and Moroc9-75, respectively, in at least one of the three time points [12]. Abebe et al. [13] studied the gene expression in the lemma, palea, awn, and seeds of barley under drought stress. The authors reported that there were only 16 upregulated and 15 downregulated genes common to all stressed organs (*q* < 0.01, |log2-fold| value ≥ 2). According to the literature review, the number of DEGs was mainly a function of the plant part used. Transcriptome data analysis in *H. vulgare*, *A. thaliana* [27, 35, 36], and many other plants shows that tolerance or susceptibility to environmental stresses is mainly controlled at the level of transcription through the interaction between transcription factors in the regulatory networks. Transcription factors are master regulators that integrate, balance, and coordinate hormonal, developmental, and environmental signals in the plant systems [37]. According to the results of the present study (Supplementary Tables 3 and 4), *HvWRKY42*, *HvEIL1*, *HvCBF6 (DREB1A)*, *DRF1.3 (DREB2A)*, *HvNAC6 (ATAF1)*, *HvERF4*, *HvFUS3*, *HvCO5 (HD1)*, and *HvVP1* (ABI3) genes were among the hub genes of the PPI networks in the vegetative and reproductive stages. These genes were activated in response to drought stress (Table 4) and have been previously reported to be related to drought-stress response [17, 25, 36, 38–40].

**Table 4:** The transcription factors which have been identified as hub genes

Barley Uniprot ID	Barley gene name	Arabidopsis orthologs	Gene family	Algorithm	Tissue
B2KJ82	*HvWRKY42*	*WRKY70*	WRKY	MNC	Reproductive stage
Q3T5P4	*HvCBF6*	*DREB1A*	AP2	MNC, Degree, Closeness	Reproductive stage
Q84JE9	*DRF1.3*	*DREB2A*	AP2	MNC	Reproductive stage
E3VXB5	*HvEIL1*	*EIL1*	EIN3 family	MNC, Degree, Closeness	Vegetative stage
F2DPJ4	*HvNAC6*	*ATAF1*	NAC	MNC	Vegetative stage
F2D6Z2	*HvERF4*	*ERF4*	AP2	MNC	Vegetative stage
B1V8R3	*HvFUS3*	*FUS3*	AP2	Closeness	Vegetative stage
Q8LGM4	*HvCO5*	*HD1*	Zinc-finger	Closeness/MNC	Vegetative stage/reproductive stage
Q8GV44	*HvVP1*	*ABI3*	AP2	Closeness	Vegetative stage

The results of the present study confirmed that transcription factors play an important role in regulating the expression of genes that are responsible for responding to drought stress. Transcription factors, as regulators of the target gene, are involved in many biological processes such as cell cycle growth, development, and response to environmental stresses. They cause the plant to adapt to adverse environmental conditions, including drought. The most important hub genes being identified in this study were transcription factors belonging to the AP2 family such as *HvCBF6*, *HvDRF1.3*, and *VP1*. These genes are involved in activating a complex drought tolerance network in barley [41]. The expression of the *VP1* gene is stimulated by abscisic acid. Its protein product as a transcription factor regulates the expression of other genes during the middle and late stages of embryogenesis [42]. The WRKY transcription factor plays a very important role in regulating growth and response to biotic and abiotic stresses [43]. In tobacco, an increase in the expression of Tawrky gene through gene transfer caused enhancements in the plant’s tolerance to drought and salinity stresses. The tolerance increased by regulating osmotic pressure and removing active oxygen species [44]. Also, a report suggested that the WRKY transcription factor increases tolerance against abiotic stresses by regulating stomatal opening and closing [45]. The four genes (*HvWRKY42*, *HvCO5*, *HvCBF6*, and *HvDRF1.3*) were selected for evaluating at reproductive stage by real-time PCR based on three reasons: firstly, they were identified as potential hub genes which are transcription factors. Secondly, they belonged to different drought-responsive gene families such as AP2 (*HvCBF6*, and *HvDRF1.3*), WRKY (*HvWRKY42*), and bZIP (*HvCO5*). Thirdly, drought stress affects all organs and developmental stages of the plants, but the reproductive stage is a critical stage as it determines the grain yield in barley [12, 13].

Interestingly, the gene expression analysis results of four potential candidate genes, i.e., *HvWRKY42*, *HvCBF6 (DREB1A)*, *DRF1.3 (DREB2A*), and *HvCO5 (HD1)* by real-time PCR in “Yousef” (the tolerant genotype) and Morocco (the susceptible genotype) indicated that all of the selected genes were upregulated in the tolerant genotype. Promoter analysis was conducted for further evaluation of regulatory factors in relation to drought stress in the plant. The AP2 family is one of the most important families of transcription factors and showed 827 (*p* value 3.58E−09) and 83 (*p* value 0.0011513) binding sites in the promoter of the drought-responsive genes. Also, it showed 47 (*p* value 4.10E−05) and 31 (*p* value 0.00062) binding sites in the promoter of the hub genes at the vegetative and reproductive stages, respectively. AP2 is one of the largest families of TFs being specific to the plant world [41]. The family has four subfamilies including AP2 (Apetala2), RAV, ERF, and DREB [46]. Among these, the subfamilies of DREB transcription factors often have a higher frequency in response to different stresses such as drought, salinity, and rapid changes in temperature and disease, as compared to other subfamilies. Among the hub genes identified in the current research, *HvCBF6 (DREB1A)*, *DRF1.3 (DREB2A)*, *HvERF4*, *HvFUS3*, and *HvVP1* also belong to this family. The genes are known to activate the complex network of drought tolerance in the plant [41]. The overexpression of DREB/CBF genes in wheat and barley lead to an increase in plant tolerance to drought stress [47]. *HvVP1* plays a crucial function during seed maturation and germination [48]. *DRF1.3* and *HvCBF6* genes specifically interact with the *HvVP1* to regulate the ABA response. *HvEIL1* and *HvFUS3* are key components of the seed development regulatory network and play an essential role in abscisic acid (ABA) responsive regulation of genes during the middle and final stages of embryogenesis [49]. During the maturation stage, grain reserves are accumulated and desiccation tolerance is achieved. This is actively controlled at the transcriptional level, and the AFL subfamily of B3 TFs plays a principal role through modulating hormone biosynthesis (mostly ABA and gibberellins) and other transcription factors of expression or their downstream activity via PPI [48]. NAC, bZIP, HD-ZIP, MYB, Zinc Finger, Trihelix, MADS, and WRKY families were also found to have binding sites in the promoter of a majority of the drought-responsive genes. It can be justified by other reports that have shown associations between them and by the genes involved in drought tolerance [38, 50–56]. The MYB family is a diverse class of DNA-binding proteins that possess one conserved DNA-binding domain called MYB which has an important role in the regulation of gene transcription. Most MYB proteins are related to the regulation of plant responses to different stresses, hormone signaling, phenylpropanoid biosynthesis, and cell differentiation. Based on the results of the current study, the MYB family had 1484 (*p* value 0.00084) and 165 (*p* value 0.0149976) binding sites in the promoter of the drought-responsive genes, whereas 118 (*p* value 0.020252) and 50 (*p* value 0.015011) binding sites in the promoter of the hub genes at the vegetative and reproductive stages, respectively.

Alexander et al. [23] studied the function of the barley transcription factor HvMYB1 and showed that gene expression is upregulated in wild-type barley roots and leaves under drought and osmotic stress. The authors also reported that transgenic barley plants that overexpress HvMYB1 were found to be more resistant to drought, showing enhanced relative water content, reduced water loss rate, and lower stomatal conductance, as compared to the control plants. Zhao et al. [57] reported that *TaMYB31* genes have different tissue expression patterns, and based on RNA-seq analysis, it was revealed that TaMYB31 functions through the upregulation of wax biosynthesis genes and drought-responsive genes. The authors confirmed that the TaMYB31 acts as a positive regulator of drought resistance and, thus, justifies its potential application in the genetic modification of drought tolerance in crops.

NAC and bZIPs have important functions in the activation of ABA-dependent signaling pathways [53, 58–60]. These families are upregulated under drought stress and some of them can increase drought tolerance due to the development of the root system [61]. Trihelix is a family of TFs and plays different roles in regulating the optical response, as well as the response to pathogens and abiotic stresses [62]. WRKY proteins are one of the largest families of transcription factors in plants and are mainly present in different biological processes such as the response to salinity and drought stress [40]. Our results showed that the HD-ZIP family of TFs had binding sites in 1945 (*p* value 4.41E−07) and 166 (*p* value 0.0083807) drought-responsive genes, along with 72 (*p* value 0.00512) and 60 (*p* value 0.014868) hub genes at the vegetative and reproductive stages, respectively. Moreover, a cooperative module was composed of two HD-ZIP transcription factors. It was discovered in 85% of the drought-responsive promoters (*P* value 1.44434E−15, 688 matches in 212 sequence) (Table 2). Previous studies have shown that the HD-ZIP family is a group of TFs in the plant world [63]. Janiak et al. [64] studied the barley root transcriptome under mild drought stress. The authors found 88 genes from 39 families involved in transcriptional regulation in roots upon mild drought. They were comprised of 13 gene TFs from the AP2 family represented by ERFs, DREB, or the B3 domain-containing TFs, eight WRKYs, six NACs, five of the HD-domain, MYB, bHLH, and bZIP TFs. Members of this family contain a combination of a homeodomain and a leucine zipper in their structure. Although these two domains separately exist in other eukaryotes, their presence in the form of one protein is only observed in plants. The role of HD-ZIP proteins in hormonal signaling pathways or in response to different stresses such as drought has already been reported [65]. In the current research, the most common combinations of transcription factor binding sites (modules) were determined in the promoter of the drought-responsive genes and the hub genes (Tables 2 and 3). Studying different biological systems has shown that the highly delicate and specific, temporal-spatial regulation of gene expression is often achieved using cooperative models in which the expression of particular genes is regulated using the simultaneous effect of two or more transcription factors under certain circumstances [66]. Several structural and biochemical studies have shown various fundamental interactions among transcription factors and structural functions of promoter/enhancer in the coordinated regulation of eukaryotic genes [67]. These studies have proposed that cooperative regulation is the main mechanism in complex patterns of gene expression [68]. Thus, studying the modules instead of transcription factors alone can meet the complexity of the response to drought in barley.

## Conclusion

The present study provided new insights into key genes associated with the plant response to drought stress in barley. This understanding occurred through the reconstruction of the related gene network and by the identification of the hub genes. Based on the results, a major proportion of the identified hub genes was involved in regulatory processes such as the regulation of transcriptions, e.g. *HvVP1*, *HvNAC6*, *HvERF4*, *HvWRKY42*, *HvFUS3, HvCO5, HvCBF6*, and *DRF1.3*, since these are from AP2, NAC, WRKY, and Homeobox transcription factor families. Moreover, a promoter analysis revealed that AP2, NAC, bZIP, WRKY, HD-ZIP and MYB are among the most common transcription factor families having a binding site in promoters of drought-responsive and hub genes. Therefore, biological processes such as regulation and gene expression play important roles in the tolerance of barley against drought stress. These insights can provide a new foundation for future research in improving plants to obtain new drought-tolerant genotypes.

## Supplementary Information

**Additional file 1.**

**Additional file 2.**

## Data Availability

All data generated or analyzed during this study are included in this published article.

## Abbreviations

PPI: Protein-protein interaction
GEO: Gene Expression Omnibus
GO: Gene Ontology
DAA: Days after anthesis
