# Correction to: New insights on key genes involved in drought stress response of barley: gene networks reconstruction, hub, and promoter analysis

**DOI:** 10.1186/s43141-021-00158-7

**Published:** 2021-05-28

**Authors:** Seyedeh Mehri Javadi, Zahra-Sadat Shobbar, Asa Ebrahimi, Maryam Shahbazi

**Affiliations:** 1 Department of Biotechnology and Plant Breeding, Science and Research Branch, Islamic Azad University, Tehran, Iran; 2 Department of Systems Biology, Agricultural Biotechnology Research Institute of Iran (ABRII), Agricultural Research, Education and Extension Organization (AREEO), Karaj, Iran; 3 Gorgan University of Agricultural Sciences and Natural Resources, Gorgan, Iran

**Correction to: J Genet Eng Biotechnol 19, 2 (2021)**

**https://doi.org/10.1186/s43141-020-00104-z**

Following publication of the original article [1], the authors reported an error in the PDF version for Figs. 2, 3 and 4 which weren’t the latest version even though the HTML version is. The revised Figures are indicated hereafter.

**Fig. 2:**
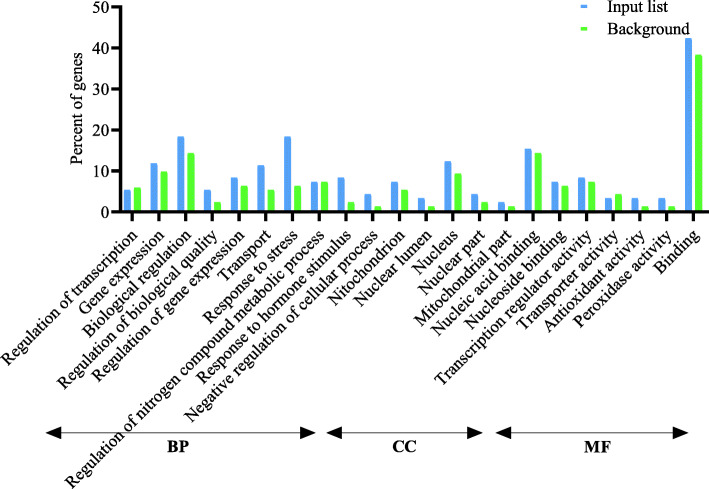
Gene Ontology enrichment analysis (BP, biological processes; CC, cellular components; and MF, molecular function) of differentially expressed genes (≤ 5 and ≥ − 5) at drought stress conditions in vegetative stage barley based on microarray data using agriGO

**Fig. 3:**
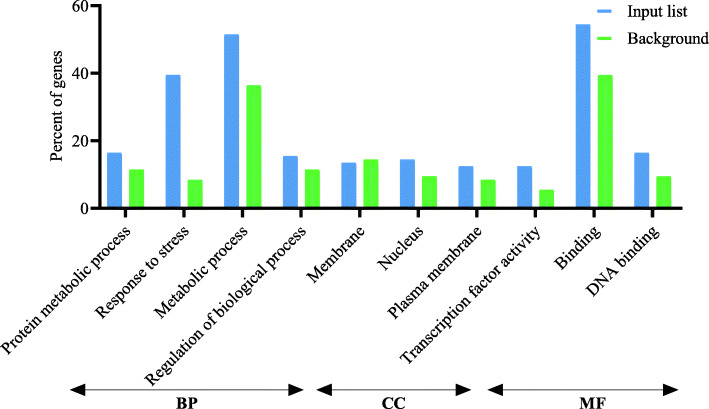
Gene Ontology enrichment analysis (BP, biological processes; CC, cellular components; and MF, molecular function) of differentially expressed genes (≤ 5 and ≥ − 5) at drought stress conditions in reproductive stage barley based on microarray data using agriGO

**Fig. 4:**
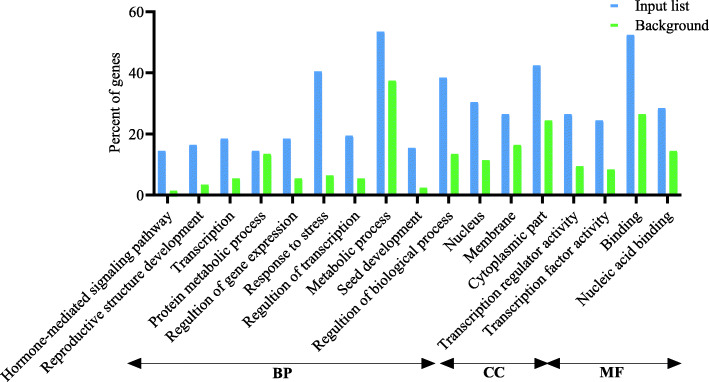
Gene Ontology enrichment analysis (BP, biological processes; CC, cellular components; and MF, molecular function) of the determined hub genes at drought stress conditions in vegetative and reproductive stage barley based on microarray data using agriGO

The changes requested are implemented in this correction and the original article [1] has been corrected.
