# Supplementary material for: New insights on key genes involved in drought stress response of barley: gene networks reconstruction, hub, and promoter analysis
Source: J Genet Eng Biotechnol. 2021 Jan 6;19:2. doi: 10.1186/s43141-020-00104-z (PMC7788114; doi:10.1186/s43141-020-00104-z)
Supplement: Supplementary file 2 — Additional file 2. [file 43141_2020_104_MOESM2_ESM.docx]

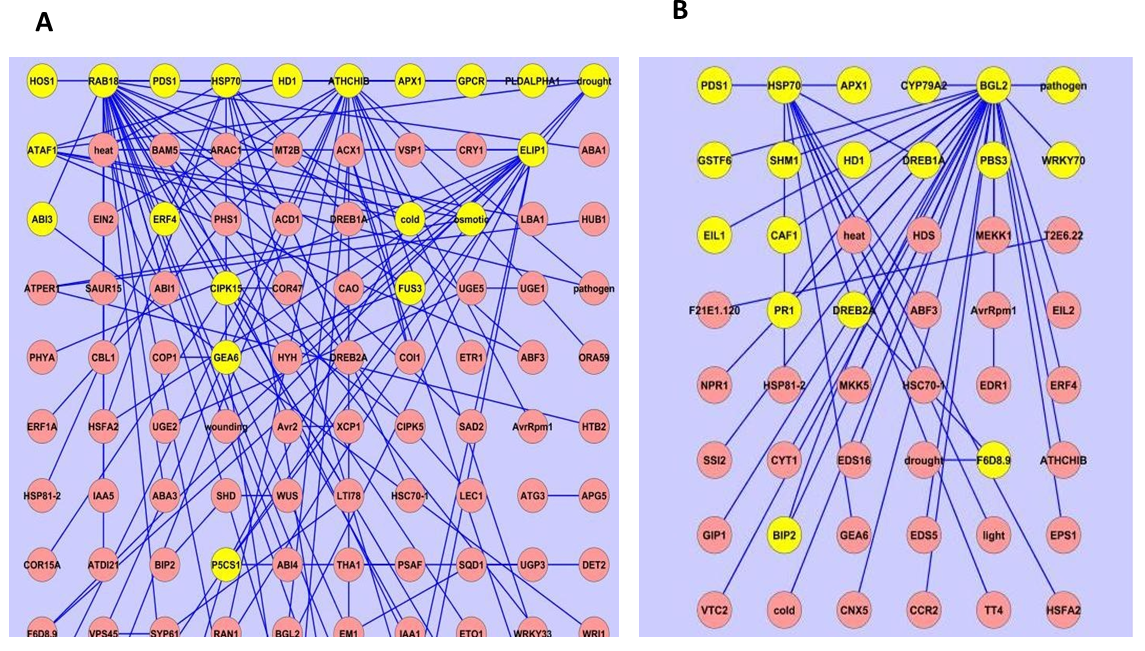


**Supplementary Fig 1:** Protein**–**protein interaction network (PPIs) for the genes involved in response to drought stress with differential expression of ≥ 5 and -5 ≥ in **A:** vegetative stage and **B:** reproductive stage of Barley based on microarray data of related hub genes drawn using Cytoscape software
